# Causal relationship between insulin resistance and sarcopenia

**DOI:** 10.1186/s13098-023-01022-z

**Published:** 2023-03-15

**Authors:** Zi-jian Liu, Cui-feng Zhu

**Affiliations:** 1 Shenzhen Clinical Medical College, Southern Medical University, Guangdong, 518101 China; 2 Shenzhen Hospital of Southern Medical University, Guangdong, 518101 China

**Keywords:** Insulin resistance, Skeletal muscle, Protein, Mitochondria, Lipid infiltration, Autophagy

## Abstract

Sarcopenia is a multifactorial disease characterized by reduced muscle mass and function, leading to disability, death, and other diseases. Recently, the prevalence of sarcopenia increased considerably, posing a serious threat to health worldwide. However, no clear international consensus has been reached regarding the etiology of sarcopenia. Several studies have shown that insulin resistance may be an important mechanism in the pathogenesis of induced muscle attenuation and that, conversely, sarcopenia can lead to insulin resistance. However, the causal relationship between the two is not clear. In this paper, the pathogenesis of sarcopenia is analyzed, the possible intrinsic causal relationship between sarcopenia and insulin resistance examined, and research progress expounded to provide a basis for the clinical diagnosis, treatment, and study of the mechanism of sarcopenia.

## Introduction

Sarcopenia refers to the phenomenon that the quality and function of muscles decrease with age, and it is an important factor leading to disability, death, and other diseases [77]. Its incidence varies widely depending on the means and criteria assessed [24]. Epidemiological surveys in Asia have found that the prevalence of sarcopenia is between 5.5% and 25.7%, with a higher prevalence in men (5.1–21.0%) than in women (4.1–16.3%) [12]. According to a global epidemiological survey conducted in 2022, the prevalence of sarcopenia is approximately 10% [80]. A large amount of clinical evidence has demonstrated that diseases of various systems of the body such as liver diseases (e.g., cirrhosis and hepatic encephalopathy), kidney diseases, endocrine-related diseases (e.g., diabetes), heart diseases (e.g., heart failure), and cerebrovascular diseases (e.g., cerebral infarction) exhibit a correlation with sarcopenia [29]. Therefore, sarcopenia has become a critical health problem threatening life and health.

**Fig. 1:**
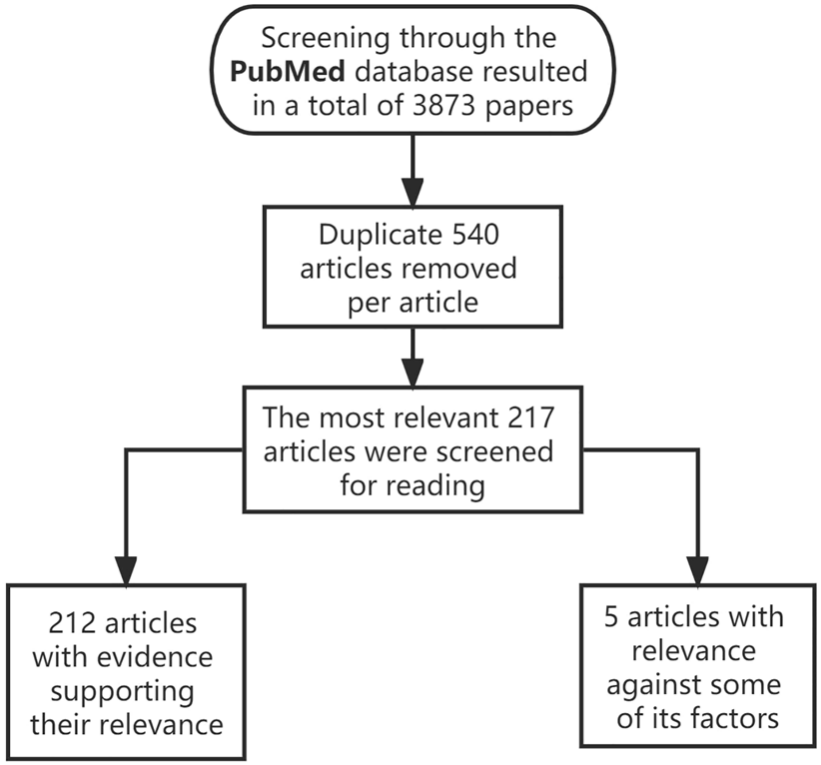
A PubMed search using the following keywords yielded a total of 3873 results. "(Insulin resistant) AND (Sarcopenia)", "Insulin amino acid skeletal muscle (() AND ()) AND ()", and "(Insulin) AND (skeletal muscle) AND (fat)". After screening, 540 repeated papers were excluded, 217 papers with the highest correlation were screened for literature reading, and experiments and treatises with high reliability were selected for citation

At present, no international consensus has been reached regarding the causes of sarcopenia. Existing studies suggest that many factors such as malnutrition, cardiopulmonary disease, endocrine diseases (such as androgen deprivation and decreased thyroid hormone levels), drugs, and congenital inheritance can cause sarcopenia. In recent years, the relationship between insulin resistance and sarcopenia has become a relatively novel hot topic. Considerable epidemiological evidence has indicated a significant association between the two [24]. However, existing literature does not specifically elaborate on this complex association. In this paper, the pathogenesis of sarcopenia is analyzed, the possible intrinsic causal relationship between insulin resistance and sarcopenia examined, and research progress expounded to provide a basis for the clinical diagnosis, treatment, and study of the mechanism of sarcopenia (Fig. 1).

## Insulin resistance is an important pathogenesis mechanism of induced muscle attenuation

The main target organ of insulin is the skeletal muscle, which accounts for 40–50% of lean body mass in adults. Glucose uptake and metabolism by skeletal muscle cells play a major role in glucose regulation [11]. Insulin resistance (IR) refers to the decreased responsiveness of the target cells of the skeletal muscle to insulin and to impaired glucose metabolism in cells throughout the body,it further induces the development of sarcopenia. Thereby, insulin resistance has been confirmed to play an important role in sarcopenia pathogenesis by inducing muscle attenuation, mainly through the following mechanisms: (1) increased protein catabolism and decreased protein synthesis in the skeletal muscle; (2) increased expression of the FoxO family, which attenuates skeletal muscle either directly or by increasing protein degradation; and (3) autophagy in skeletal muscle cells.

### Insufficient supply of amino acids and decreased sensitivity of insulin receptors together contribute to the development of sarcopenia

Muscle attenuation is associated with inadequate intake of amino acids and decreased insulin sensitivity. On the basis of several experimental reports and findings, we propose two complete signaling pathways, the insulin signal transduction pathway and amino acid signal transduction pathway; we detail the mechanism by which amino acids act in combination with insulin to promote an increase in skeletal muscle protein and emphasize the role of key factors.

The decrease in muscle mass is caused by an imbalance between the rate of protein synthesis and the rate of protein breakdown in the muscle [1, 95]. Through studies in elderly subjects, Fujita et al. found that insulin plays an important role in promoting the synthesis of muscle proteins and inhibiting muscle protein catabolism in humans [39]. An earlier study of patients with diabetes has reinforced this view: Atchley et al. found that patients with type 1 diabetes mellitus along with absolute insulin deficiency showed a series of changes such as increased urinary nitrogen, increased free amino acids, and increased amino acid breakdown, which can largely reverse these clinical manifestations when exogenous insulin is infused [7]. In a recent study, Makanae et al. observed that after exogenous injection of insulin, the concentration of free essential amino acids in the human body decreased, while the synthesis of muscle proteins increased [69]. Additionally, by reviewing the effects of amino acid-induced protein synthesis in neonatal skeletal muscle, Suryawan et al. confirmed that amino acids can act as independent influencing factors to stimulate muscle protein synthesis [100] and that the supply of abundant amino acids can not only inhibit protein hydrolysis but also increase protein synthesis. In healthy humans, insulin and amino acids act synergistically to promote muscle protein synthesis.

#### AKT is the first key molecule in insulin signaling

Insulin receptors (INSR) are classified into IRS1 and IRS2, which play a major role in the growth and development of skeletal muscle. Long et al. used creatine kinase to block IRS1 expression in mouse skeletal muscle and found a decrease in skeletal muscle mass and skeletal muscle cell insulin sensitivity; however, blocking IRS2 did not produce the same results. However, simultaneous blockade of IRS1 and IRS2 leads to severe skeletal muscle attenuation and decreased activation of the insulin signaling pathway, suggesting that the two produce a synergistic effect in the skeletal muscle [65]. Following insulin activation of IRS1, IRS1 continues to activate its downstream phosphatidylinositol-3 kinase (PI3K). PI3K can catalyze phosphatidylinositol 4,5-bisphosphate (PIP2) to produce phosphatidylinositol 3,4,5-triphosphate (PIP3) [101], and the reverse reaction is catalyzed by phosphatase and tensin homologues deleted on chromosome 10 (PTEN). Insulin can inhibit PTEN activity, thereby increasing the levels of PI3K catalysis products [104]. PIP3 recruits proteins with PH domains to the lipid membrane and facilitates the localization of the downstream signaling factors phosphoinositide-dependent kinase 1 (PKD1) and AKT. In addition, PIP3 accumulation can transmit downstream and amplify insulin signaling.

AKT can inhibit its downstream TSC complex in the muscle, and this molecule limits Rheb activity, resulting in the inhibition of mTORC1 activity, as observed by Dibble et al. Therefore, AKT can increase MTORC1 activation levels by inhibiting the TSC complex. Although insulin stimulates AKT activation, the pathway by which insulin stimulates AKT Ser473 activation remains unknown. Dong et al. found that insulin stimulated phosphorylation of AKT Ser473 in IRS1-deficient and IRS2-deficient mice in animal experiments [33], but the level of AKT phosphorylation decreased to some extent

TORC has two isoforms, and although mTORC2 is not directly associated with amino acid infusion and protein synthesis, it is similarly stimulated by growth factors such as insulin. PIP3 is produced after PI3K activation by insulin, and PIP3 activates Akt while also activating mTORC2. Pearce et al. demonstrated that although PIP3 can directly activate Akt, Akt requires additional phosphorylation of its carboxyl terminal site S473 by mTORC2 to achieve the highest level of activation; phosphorylation at this site can increase Akt activity by more than several folds. In addition, mTORC2 plays a role in stabilizing Akt conformation [78]. Thus, substantial experimental evidence has confirmed that mTORC2 plays a crucial role in the PI3K–Akt–mTORC1 pathway and that it is required at all levels of signaling.

#### mTORC1 is the second key molecule in insulin signaling

When activated, Rheb can stimulate the activity of the downstream mTORC1 kinase [35, 92]. PRAS40, an inhibitory subunit of mTORC1 and a substrate of Akt, can be phosphorylated by both mTORC1 itself and Akt, causing the dissociation of PRAS40 from mTORC1 and further enhancing the effect of mTORC1 on its downstream substrates. When mTORC1 is activated, it senses nutrients such as amino acids through the Rag GTPase pathway. Rag GTPases do not activate mTORC1 by themselves; however, they can promote their tropism towards Rheb in cells.mTORC1 activation promotes protein synthesis mainly by phosphorylating various substrates downstream of mTORC1, such as activating S6K1 and inhibiting 4EBP1 [14]. Timmerman et al. found in human experiments that the effect of mTORC1 on these two substrates can increase the expression level of the initiation factor of the two mRNAs translated by phosphorylated proteins to promote protein synthesis [102]. Defects in mTORC1 activation lead to decreased protein synthesis, as well as resistance to growth and metabolism factors such as insulin [22]. Previous studies have shown that S6K1 can promote translation and peptide chain elongation by phosphorylating its downstream substrates, such as ribosomal protein S6 and the protein synthesis initiation factor 4B (elF4B) [5]. The insulin action pathway inhibits protein breakdown by inhibiting the FOXO family while activating mOTRC1 [36],we will focus on this issue in the next section.

Through negative feedback, S6K1 can regulate protein synthesis pathways by degrading IRS1; however, this effect is maintained at very low levels in normal physiological states. Barbour et al. found that long-term increases in S6K1 lead to degradation of the insulin receptor IRS1 in patients with impaired glucose tolerance postpartum [10],this effect may be more important for maintaining life because it reduces amino acid uptake and protein synthesis in the skeletal muscle, in turn allowing more amino acids to synthesize plasma proteins in important organs such as the liver. Regulation of insulin sensitivity is a complex and dynamic physiological process. Here, we focus on describing its interactions with amino acids and effects associated with increasing skeletal muscle mass.

Kim et al. found that another important factor in the insulin action pathway is AMP-activated protein kinase (AMPK), which can activate TSC2; transgenic induction of the gene expression of TSC2 led to decreased activation of mTORC1 in Drosophila. TSC2 antagonizes the physiological effects of insulin, reducing protein synthesis and increasing autophagy levels [54].

Many studies have shown that insulin and amino acids together promote increased protein synthesis and thereby muscle mass, while amino acid and insulin action ultimately play a synergistic role in activating mTORC1. Arguably, mTORC1 unites two separate pathways of action. To confirm that mTORC1 is a key node in activating protein synthesis, Dickinson et al. designed an experiment in which subjects in the experimental group ingested 10 g of essential amino acids; muscle biopsy results confirmed a 60% increase in muscle protein synthesis levels. In control group subjects, who ingested amino acids after the use of mTORC1 inhibitors, muscle biopsy showed little change in muscle protein synthesis levels. This study strongly confirmed the key role of mTORC1 in the insulin–amino acid costimulatory pathway [31]. In addition, in a review, Ruegsegger has detailed several experimental conclusions indicating that the specific deletion of mTORC1 leads to high muscle atrophy and early death in mice [88]. Compared with healthy individuals, patients with insulin resistance lack the specific inhibitory effect of Akt on the TSC complex, show higher levels of TSC1/2 activation, and exhibit greater mTORC1 inhibition [26], resulting in decreased levels of amino acids and protein synthesis in the muscle. Therefore, the quality of the muscle decreases, causing the development of sarcopenia.

However, insulin does not stimulate an increase in the synthesis of all proteins, and the sensitivity of different proteins to its stimulatory effects is different: for example, myosin heavy chain synthesis is not directly associated with insulin levels. An important effect of insulin on cells is maintaining the function of mitochondria, and insulin stimulation of mitochondrial protein synthesis is important for maintaining the normal metabolism of skeletal muscle cells.

Insulin can regulate mitochondrial function through the mTORC and FoxO transcription factor pathways because mTORC and FoxO1 can regulate PPAPγ activity factor 1α [17]. PGC1A controls mitochondrial gene transcription through nuclear respiratory factors 1 and 2 (NRF1/2) and the nuclear transcription factor TFAM. Studies have shown that after insulin injection, increased levels of insulin can increase the levels of mRNA for nuclear genes encoding mitochondrial proteins (cytochrome c oxidase subunit IV) and mitochondrial genes, such as NADH dehydrogenase subunit IV (MT-ND4), and can promote the synthesis of mitochondrial proteins [44]. Zhao et al. performed muscle biopsies 4 h after exogenous insulin infusion in healthy subjects,the results showed a two-fold increase in the number of phosphoproteins in isolates of mitochondria. Further analysis of the components of mitochondrial isolates revealed a substantial increase in the number of proteins associated with mitochondrial function, such as those responsible for mitochondrial transport and the TCA cycle [86]. Normal levels of mitochondrial protein synthesis are important for maintaining mitochondrial function [88, 98]. Scientists have found that mitochondrial activity is dramatically reduced in patients with congenital defects in insulin receptor signaling. In addition, elderly patients with insulin resistance show decreased insulin stimulation of mitochondrial protein synthesis and enzyme activity, resulting in decreased mitochondrial function. Lower mitochondrial oxidative capacity leads to fat accumulation within the skeletal muscle, leading to further development of insulin resistance [22, 51].

**Fig. 2:**
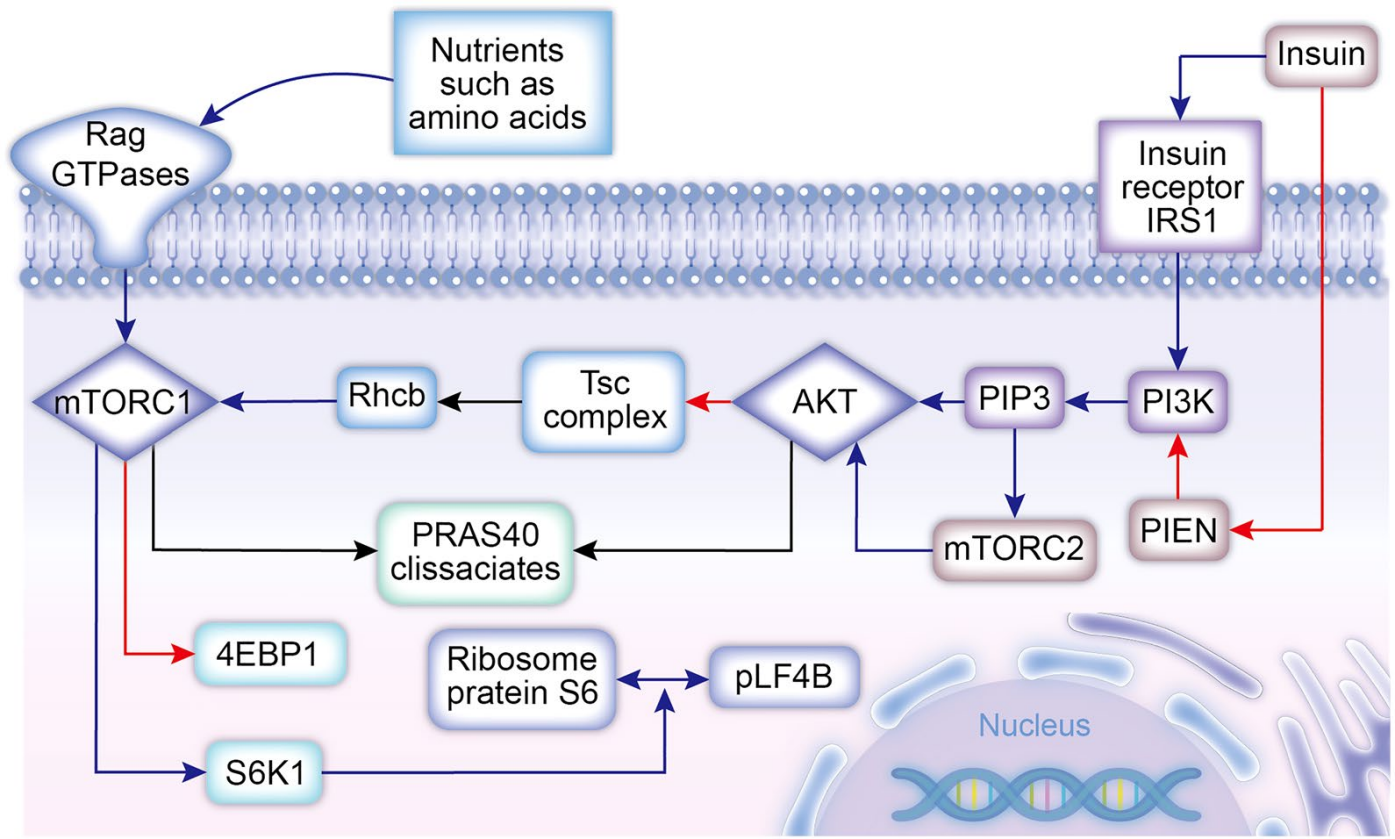
Cell stimulation by insulin and amino acids is the main factor that affects intracellular protein synthesis and increases intracellular perfusion of amino acids. Amino acids and insulin increase the activation level of mTORC1 via different pathways. Insulin relieves the inhibitory effect of the TSC on mTORC1 primarily by increasing the activation level of AKT, while amino acids can directly activate mTORC1. An increase in the activation level of mTORC1 subsequently promotes the synthesis of myocyte proteins. Red arrows represent inhibitory effects, and blue arrows represent activating effects. *PIP3* phosphatidylinositol 3,4,5-triphosphate

A large body of experimental evidence indicates that sufficient amounts of available amino acids are initiators, which recruit large amounts of mTORC1 [90], while insulin activates mTORC1 in response to a series of downstream pathways. This ultimately promotes increased amino acid perfusion and protein synthesis, both of which together maintain the quality and quantity of skeletal muscle cells (Fig. 2).

### FOXO family induces skeletal muscle attenuation directly or by increasing protein degradation

In this section, we focus on the FoxO-family-regulated protein breakdown pathway. A large increase in protein breakdown is an important cause of muscle attenuation, and several studies have confirmed that insulin mainly maintains the number of skeletal muscle cells and skeletal muscle mass by inhibiting protein catabolism.

The most important function of the FoxO family is to regulate protein catabolism levels. In eukaryotic cells, most proteins are degraded through the ubiquitin–proteasome [60] and autophagy–lysosomal [66] pathways. The FoxO family can affect skeletal muscle atrophy by regulating factors related to the cellular proteolytic system; for example, nuclear translocation of FoxO can activate atrophy genes to activate these two pathways [16]. FoxO is a downstream factor of Akt, and elevated Akt levels can significantly reduce FoxO levels in the cytoplasm and gene expression in the nucleus [61]. T Through the effects of Akt, insulin can indirectly inhibit FoxO activity [20]. Price et al. demonstrated increased protein degradation in insulin-resistant mice, whereas treatment with the insulin receptor sensitizer rosiglitazone partially ameliorated the higher levels of degradation [82].

Different isoforms of FoxO play a role in sarcopenia through different pathways. FoxO1 can inhibit mTORC levels, which leads to elevated levels of myostatin and, consequently, abnormalities in myogenic differentiation [107]. Therefore, FoxO1 overexpression can directly cause the development of muscle attenuation [49, 91]. FoxO3 contributes to massive degradation of skeletal muscle proteins through massive activation of autophagy and ubiquitin proteases, such as atrogin-1, MuFR1, and TRIM63 [71]. FoxO4 inhibits the proliferation of muscle progenitor cells, which leads to muscle atrophy [94]. In mice, knockout of the FoxO trigene resulted in a significantly decreased level of muscle attenuation and wide inhibition of the ubiquitin–proteasome and autophagy–lysosomal pathways, indicating that the FoxO family is an important mediator of muscle attenuation. Wei et al. recently demonstrated in animal experiments that the levels of FoxO1, FoxO3a, MaFbx, and MuFR1 were significantly increased in mice with type 2 diabetes mellitus (T2DM). MaFbx and MuFR1 are ubiquitin protein ligases, and their increased levels reflect increased skeletal muscle protein breakdown. Following zanthoxylamide (ZA) treatment, the PI3K–Akt pathway activity increased and FoxO gene activation levels decreased [106], which further confirmed the role of the insulin-acting pathway in inhibiting FoxO-dependent protein degradation pathways. Furthermore, Milan et al. induced elevated levels of protein breakdown by starvation treatment of mice following specific knockdown of the FoxO trigene (FoxO1, 3, and 4). Experimental results showed that 26 genes associated with muscle atrophy were not expressed in mice with FoxO trigene knockout, and pathways involved in protein breakdown were completely blocked [72].

Numerous clinical experiments have demonstrated that 125 genes involved in the ubiquitin–proteasome pathway have significantly elevated gene expression levels in patients with diabetes, and this change disappears after FoxO is specifically knocked down. Among these genes, scientists have paid special attention to the levels of ubiquitin-activating enzyme (Ube4a), E2-ubiquitin-conjugating enzyme (Ubeq2q), and E3-ubiquitin ligase (Itch). O'Neill et al. used streptozotocin (STZ) to induce diabetes in mice for detection of gene expression, and the experimental results showed that Ube4a, Ube2q2, and Itch tended to gradually increase with the progression of diabetes; however, the levels of the above three genes did not change significantly in mice with FoxO gene-specific knockout [76]. The autophagy protein LC3 can reflect the activity level of autophagy–lysosomal pathway. The researchers found more than a two-fold increase in the level of LC3 per unit area and increased levels of the autophagy-inducing marker Ulk1 in patients with insulin resistance.

Additionally, experimental evidence indicates that the FoxO family can directly induce the development of sarcopenia. When cells develop insulin resistance, the activity of FoxO target genes is significantly increased, in particular, the Gadd45a gene. Ebert et al. specifically induced its overexpression in the skeletal muscle and found that Gadd45a can directly mediate atrophy of skeletal muscle [36] and cause muscle attenuation. Thus, the mechanism of action of FOXO in regulating muscle atrophy genes is very complex, and current knowledge on this topic has several gaps.

### Changes in autophagy levels in skeletal muscle cells may be one of the pathogenesis mechanisms leading to skeletal muscle attenuation

Overactivation of the autophagy pathway leading to muscle attenuation has been a hot research topic; however, considerable evidence also indicates that high expression of the autophagy pathway and reduced levels of the autophagy pathway can have different effects on insulin resistance and muscle attenuation. Therefore, its specific role needs to be further explored.

Autophagy refers to the process in which eukaryotes use lysosomes (in animals) to degrade their own cytosolic proteins and damaged organelles under the regulation of autophagy-related genes or molecules and finally perform recycling. The muscle is one of the most active organs of the autophagy pathway [2]. Two causes of autophagy in cells are inadequate nutrient supply and deficiency of growth factors (e.g., insulin and IGF). Activation of mTORC1 by insulin in humans directly phosphorylates the kinases UlK1 and ATG13, resulting in inactivation of a complex composed of UlK1 and ATG13, which thereby attenuates autophagy levels. However, another isoform mTORC2 can upregulate or inhibit autophagy in different physiological environments and exerts dual effects. Additionally, insulin can negatively regulate autophagy by disrupting the transcription factor EB (TFEB).

We have discussed in the previous section that insulin can inhibit the FoxO family, resulting in decreased levels of the autophagy–lysosomal pathway. The FoxO family, especially FoxO3, is involved in the induction of the autophagic ligase FBXO32. Seri et al. induced muscle attenuation in mice by knocking out arginine methyltransferase 1 (PRMT1) and found that transcripts of FoxO3 were expressed by more than two-fold in mice with muscle attenuation. Additionally, immunostaining showed that more FoxO3-positive myonuclei were found in the mouse muscle than in healthy mice from controls and that even the central nucleus of muscle fibers with low distribution increased by different levels in different physiological states, which was positively correlated with the increase in autophagy levels [21]. Upregulation of the autophagy pathway can be blocked by knocking down FoxO genes or enhancing Akt gene expression. Numerous animal experiments have demonstrated that starvation and lack of growth factors can lead to overexpression of the autophagy pathway, resulting in the development of sarcopenia. However, the observations of Yamamoto et al. in animal experiments were different from the above results; the team found that high levels of autophagy activation in the skeletal muscle may improve insulin resistance by downregulating ER stress response levels [108].

More worthy of our in-depth consideration are the experiments of Kim et al. ATG7 is a key protein in autophagosome formation, and the team found that mice with specific deletions in the ATG7 gene developed severe muscle atrophy; however, these mice showed higher resistance to diet-induced insulin resistance. Attenuation of autophagy may preserve insulin sensitivity, possibly because upregulation of FGF21 actin levels promotes insulin sensitivity [53]. However, it is unclear whether muscle atrophy occurs while maintaining insulin sensitivity.

In recent years, many experiments have shown that decrease in autophagy pathway activity can lead to the aggravation of insulin resistance. Frendo-Cumbo et al. found that the knockdown of Atg16l1 in L6 myocytes leads to a decrease in autophagy levels, while the level of IRS1 is significantly reduced [81]. Recently, scientists have proposed a new mechanism by which autophagy may affect insulin resistance, that is, long-term insulin resistance leads to excessive activation of mTORC1, which leads to extreme downregulation of the level of autophagy and further promotes the development of insulin resistance and sarcopenia.

Our review of the literature indicates that, according to experimental evidence, both the rise and fall in autophagy levels have demonstrated an inverse association with insulin sensitivity, which may require further investigation.

Insulin resistance can lead to the occurrence of sarcopenia through many pathways, and the above mechanisms of action are popular topics for in-depth research. The PI3K–AKT–mTORC pathway as the most important mechanism underlying this effect builds a bridge between the skeletal muscle and insulin, and a series of pathways such as FOXO and autophagy can be regulated through this pathway. By continuously exploring the downstream targets of each protein, we can continuously discover new sites to identify new treatment strategies for insulin resistance and sarcopenia. In recent years, an increasing number of downstream factors have been discovered, which will be a hot field of future research.

## Sarcopenia is an important mechanism underlying the development of insulin resistance

Numerous studies have confirmed that sarcopenia can induce insulin resistance through: (1) abnormal lipid infiltration distribution and lipotoxicity in the skeletal muscle, (2) increased amino acid catabolites in skeletal muscle branch chains, and (3) decreased proportion of skeletal muscle type I fibers.

### Abnormal fat distribution and infiltration in the skeletal muscle can lead to insulin resistance through multiple pathways

Abnormal fat production and intracellular lipid deposition in the skeletal muscle are among the most important factors causing insulin resistance. In this section, we review a large number of studies that show that lipid infiltration can cause the development of insulin resistance through several different mechanisms.

#### Abnormal distribution and number of adipocytes and the occurrence of inflammation can cause insulin resistance

Studies have shown that with the progression of age, human body fat will undergo proliferation, hypertrophy, and redistribution, and considerable fat infiltration will occur in the skeletal muscles, leading to myosteatosis. Myosteatosis is different from obesity, and the occurrence of both obesity and sarcopenia is called obesity-associated sarcopenia (SO). Adipocytes exist in three forms in the muscle, namely, intermuscular adipose tissue, intramuscular adipose tissue, and intramyocellular adipose tissue. Studies have shown that a large part of lipid infiltration occurs in the cytoplasm. Myocyte lipids play an important role in inducing insulin resistance, and many lipid intermediates affect insulin signaling, resulting in lipotoxicity. Intramuscular lipids (IMCLs) exist as lipid droplets (LDs) surrounded by members of the periphospholipid protein (PLIN) family, among which the bone expresses mainly PLIN2, 3, and 5. PLIN2 maintains the stabilization of lipid droplets and prevents degradation, which is a main factor limiting IMCL deposition. de Wilde et al. demonstrated through animal experiments that upregulation of lipotropic proteins such as PLIN2 is associated with improved insulin sensitivity [28]. In this section, we focus on lipids that are strongly associated with insulin resistance.

Diacylglycerol (DAG) exists as three isomers in the muscle, of which 1,2-DAG forms the vast majority, and it is the only one that has been demonstrated to negatively correlate with insulin sensitivity in humans. Accumulation of DAG in cells leads to activation of isoforms of protein kinase C (PKC), the most important of which is PKCθ. PKCθ in the muscle leads to inhibition of ISR1, leading to the development of insulin resistance [56]. However, Perreault demonstrated through human experiments that sarcolemmal DAG was higher in athletes, compared with that in lean individuals, similar to that in patients with T2D and those with obesity. This is possibly because difference in the proportion of DAG to other lipids or deposition in different organelles or cytosol resulted in completely different results. Subcellular localization of lipids is closely related to insulin resistance [79],for example, the content of 1,2-DAG in the mitochondria and endoplasmic reticulum is positively correlated with insulin sensitivity, which may be because 1–2-DAG increases mitochondrial cristae density and oxidative capacity. Nielsen et al. showed experimentally that the density of mitochondrial cristae in athletes was higher than that in normal individuals [74]. Additionally, the team has demonstrated in animal experiments that elevated DAG levels lead to enhanced oxidative capacity of mitochondria [74].

Ceramide (CER) can be transported intracellularly by transport proteins (CERT), which are significantly increased in patients with obesity. Accumulation of CER leads to decreased insulin sensitivity and decreased activity of Akt, probably because activation of protein phosphatase 2A leads to inhibition of dephosphorylation of Akt [18, 19]. In addition, CER may alter the activity of complexes 1 and 3 in the respiratory chain, membrane structure, and permeability, resulting in inhibition of respiration in mitochondria [30, 42]. Unlike DAG, a large accumulation of CER in the mitochondria and endoplasmic reticulum leads to a decrease in insulin sensitivity.

Acylcarnitine (ACC) accumulates when lipids are incompletely oxidized, which leads to the development of insulin resistance [47, 57]. Both medium-chain and long-chain ACC have been shown to be associated with insulin resistance, and Nowak et al. showed through human experiments that the accumulation of C-10 and C-12 carnitine in medium-chain ACC reduces insulin sensitivity in mouse adipocytes [75],however, the specific mechanism of action has not been demonstrated. Liepinsh et al. found a decrease in insulin sensitivity in mouse myocytes after administration of palmitoylcarnitine (PC) in mice, and further studies showed that long-chain acylcarnitine could reduce the phosphorylation level of Akt Ser473 to induce insulin resistance [64]. In addition, accumulation of ACC leads to decreased function of mitochondria and increased apoptosis, leading to insulin resistance [73].

In addition to adipocytes themselves affecting insulin sensitivity through multiple pathways, lipid infiltration of myocytes leads to massive activation of proinflammatory factors, such as tumor necrosis factor-α (TNF-α), monocyte chemoattractant protein (MCP-1), and interleukins. MCP-1 recruits monocytes to sites of inflammation [37, 50], and monocytes transform into macrophages once monocytes at sites of inflammation bind to adipocytes. In normal adipose tissue, macrophages are phenotyped as M2, which exerts anti-inflammatory effects, whereas M2 macrophages in inflamed adipose tissue are largely polarized to M1 macrophages. M1 macrophages are pro-inflammatory, and they release multiple inflammatory factors that in turn recruit more monocytes [3], leading to a vicious cycle. TNF-α promotes the activity of a series of proteins such as IKK that act directly on the substrate (IRS) of the insulin receptor, leading to the development of insulin resistance in muscle cells [4, 83]. In addition, TNF-α can inhibit phosphorylation of AMPK Thr172 via the action of protein phosphatase 2C (PP2C), leading to increased levels of free fatty acids and a tendency towards insulin resistance [9, 87, 97]. IL-6 induces activation of the JAK-STAT pathway and increases the expression of suppressor of cytokine signaling 1 and 3 (SOCS1 and SOCS3), leading to decreased sensitivity of the insulin receptor [84, 99].

#### Lipotoxicity causing damage to organelles is an important factor leading to insulin resistance

The mitochondria and endoplasmic reticulum are important organelles for maintaining normal metabolism in myocytes. In this section, we focus on the effects of lipotoxicity on these organelles.

The infiltration of lipids into myocytes results in the production of large quantities of reactive oxygen species (ROS), which opens the mitochondrial membrane permeability pores (PT pores) and induces the release of calcium ions, cytochrome C, and the apoptosis-inducing factor (AIF). These events subsequently activate caspase 9, which leads to the uncoupling of the mitochondrial electron transport chains. This leads to an increase in the expression of the pro-apoptotic protein Bax and induces the rupture of the mitochondrial outer membrane, resulting in apoptosis. A large number of damaged mitochondria are removed by autophagy in the body [105], causing a decrease in mitochondrial density and function, impairing the oxidative capacity of skeletal muscle cells, further leading to lipid infiltration, ectopic lipid deposition, and progression of insulin resistance [25]. Espinosa et al. found increased release of H_2_O_2_ in high-fat diet (HFD)-fed mice [38]. When lipid deposition is excessive, to compensate, myocytes alter their own partitioning of energy metabolism by increasing the relative lipid utilization. When lipid utilization increases, no corresponding substrate is available for the ATP synthesis pathway, which limits lipid utilization by cells and ultimately leads to IMCL deposition. At the same time, such compensatory mechanisms lead to increased fatty acid β oxidation (FAO) and increased ROS production, further leading to IMCL deposition and the development of skeletal muscle insulin resistance, forming a vicious cycle. Holmström et al. have demonstrated in animal experiments that mitochondrial dysfunction and increased levels of apoptosis in the skeletal muscle of obese mice are directly related to the development of skeletal muscle insulin resistance [43].

Mitochondrial proton leak refers to ATP synthesis by mitochondria in the absence of phosphorylated ADP. Protons (H^+^) do not face the action of ATP synthase but return to the matrix directly through the inner mitochondrial membrane. Normally, state 3 respiration and state 4 respiration of the mitochondria are performed alternately; therefore, electron leakage from the mitochondria is the main source of ROS. When respiratory chain activity is blocked, ROS levels rise, which has been confirmed in clinical studies of patients with T2DM.

In 2000, Shusen proposed the "mechanism of proton leak caused by electron leak and the circulation theory of reactive oxygen species", which suggests that ROS produced by electron leak can cause proton leak as endogenous carriers of H^+^. Mitochondrial proton leak can be concluded to be an important mechanism by which the body removes ROS and protects itself. Kenny et al. showed a marked decrease in uncoupling levels in skeletal muscle mitochondria, that is, a decrease in proton leak from the mitochondria, measured after 21 days of bed rest. The long-term decrease in LEAK respiration is closely related to the rise in ROS levels and increase in oxidative damage, which may be due to decreased levels of ANT1/ANT2 [52]. ANT1/ANT2 plays an important role in mitochondrial proton leak kinetics,however, its mechanism of action needs to be explored.

A large amount of experimental evidence has demonstrated that the most relevant increase in ROS levels in various types of lipids is the accumulation of CER, which can lead to a decrease in state 3 respiration and increase in state 4 respiration, resulting in an increase in ROS levels. However, views on mitochondrial coupling reactions have been inconsistent in different studies. For example, patients with type 1 diabetes have elevated levels of systemic oxygen consumption but decreased ATP production, which represents increased levels of mitochondrial uncoupling, that is, insulin resistance leads to massive production of heat energy, while ATP production is reduced. Stanford et al., therefore, considered that a mild induced rise in uncoupling could serve as a new strategy in insulin resistance therapy; however, a significant rise in uncoupling could be life-threatening [96].

Mitochondrial structure and function are maintained through a series of fusion division activities. The fusion of outer membranes during mitochondrial fusion is controlled by mitofusion proteins 1 and 2 (Mfn1/Mfn2), and obesity downregulates Mfn2 gene expression [58]. The key proteins of mitochondrial division are mitochondrial dynamin-associated protein (Drp1) and recombinant human mitochondrial division protein 1 (Fis1). HFD leads to increased Drp1 and Fis1 expression, resulting in increased mitochondrial breakdown in the skeletal muscle, which causes the development of insulin resistance. Additionally, studies have demonstrated that patients with obesity and T2DM exhibit higher skeletal muscle Drp1 protein content than healthy individuals. Impaired mitochondrial dynamics combined with increased lipid deposition leads to impaired lipid droplet turn over, such as abnormal intracellular distribution of PLIN2 necessary for lipid titration, which may lead to increased ectopic deposition in IMCL, thereby exacerbating ROS accumulation and the degree of insulin resistance [55]. Furthermore, recent studies have shown that mitochondrial damage can promote the progression of insulin resistance through FoxO3.

Endoplasmic reticulum (ER) stress is one factor leading to insulin resistance. ER stress occurs in association with massive accumulation of lipids and lipotoxicity. To improve ER stress, the ER activates a series of signaling pathways, such as enhancing the folding ability of proteins and accelerating protein degradation, known as the unfolded protein response (UPR). ER stress is primarily caused by three transmembrane proteins, namely, inositol-requiring enzyme 1 (IRE1), activating transcription factor 6 (ATF6), and the protein kinase-like endoplasmic reticulum kinase. Among them, IRE1 can lead to JNK phosphorylation. JNK is a pro-inflammatory transcription factor whose increased levels result in decreased AKT and GSK3β levels and can directly contribute to the development of insulin resistance [62]. Scientists have found that JNK-specific deletion in the muscle significantly protects insulin sensitivity in HFD-fed mice in which insulin resistance was induced. JNK can induce the development of inflammatory responses, and a study showed that decreased levels of JNK1 resulted in significantly reduced macrophage infiltration, decreased M1 phenotype, increased M2 phenotype, and decreased levels of local proinflammatory cytokines (TNF-α and IL-6), which indicated that decreased levels of JNK1 could reduce the level of inflammation and improve insulin resistance. Li et al. demonstrated that JNK also promoted phosphorylation of IRS1 Ser307, resulting in decreased insulin receptor binding [63]. However, Sabio et al. showed in animal experiments that phosphorylation of IRS1 Ser307 at a single site is insufficient for inducing the development of insulin resistance [89],therefore, further investigation is required to determine whether JNK1 phosphorylates multiple sites to induce insulin resistance.

Interaction between the ER and mitochondria is another focus of research. Scientists have found that ER stress can cause an increase in the concentration of Ca^2+^ in the mitochondria, which causes an increase in ROS production and leads to mitochondrial damage. Additionally, overactivation of mTORC1 may be linked to ER stress. Ozcan et al. found experimentally that TCS2-specific-knockout mice exhibit profound ER stress that further aggravates insulin resistance through multiple pathways.

#### Other mechanisms associated with lipid infiltration of myocytes

Ubiquitination of IRS1 showed generally elevated levels in HFD-fed mice. E3 ubiquitin–proteasome ligases have been found to be critical in determining substrates for degradation by ubiquitination, and they can degrade IRS directly through the ubiquitin–proteasome pathway (UPS); for example, IRS1 can bind to E3 ligases such as MDM2, Cbl-b, and MDM27 and undergo degradation, thereby reducing cellular sensitivity to insulin. PDCD5, an apoptosis-accelerating protein, is widely present in the skeletal muscle, and Zhou et al. found in animal experiments that it specifically promotes ubiquitination of MDM2 itself to protect IRS1 from damage [110]. The ubiquitin–proteasome pathway can not only lead to muscle attenuation by affecting protein degradation but also lead to IRS1 degradation, affecting insulin sensitivity and forming a vicious cycle. Therefore, PDCD5 could serve as a novel target to improve insulin resistance in the skeletal muscle.

Scientists have recently explored new ways to link lipotoxicity to insulin resistance. Skeletal muscle PANX1 is a channel involved in the release of ATP into the extracellular space, and extracellular ATP (eATP) can activate many inflammatory responses [34] and participate in several pathophysiological changes [70]. Jorquera showed experimentally that eATP levels in obese mice were significantly increased compared with that in the controls and that they decreased by more than 80% after specific knockdown of PANX1, consistent with the results obtained for PANX1 inhibitors. Scientists then found that local inflammation was significantly aggravated by measuring inflammatory markers such as TNF-α, IL-4, and TOLL4, indicating that the development of eATP-induced insulin resistance may be due to several inflammatory responses [48]. Recent studies have shown that the level of Akt phosphorylation in human mature skeletal muscle cells is negatively correlated with the eATP level. In summary, obesity leads to high expression of PANX1 channels in the skeletal muscle, leading to the development of insulin resistance.

Mitogen-activated protein kinase phosphatase-1 (MKP-1), an enzyme expressed in the muscle, is abnormally elevated in the skeletal muscle of patients with obesity, and MKP-1 prevents phosphorylation of Akt Ser473 and signaling of molecules such as p38 AMPK, resulting in insulin resistance. Lawan et al. demonstrated this result in mice in which the MKP gene was specifically knocked out [59].

**Fig. 3:**
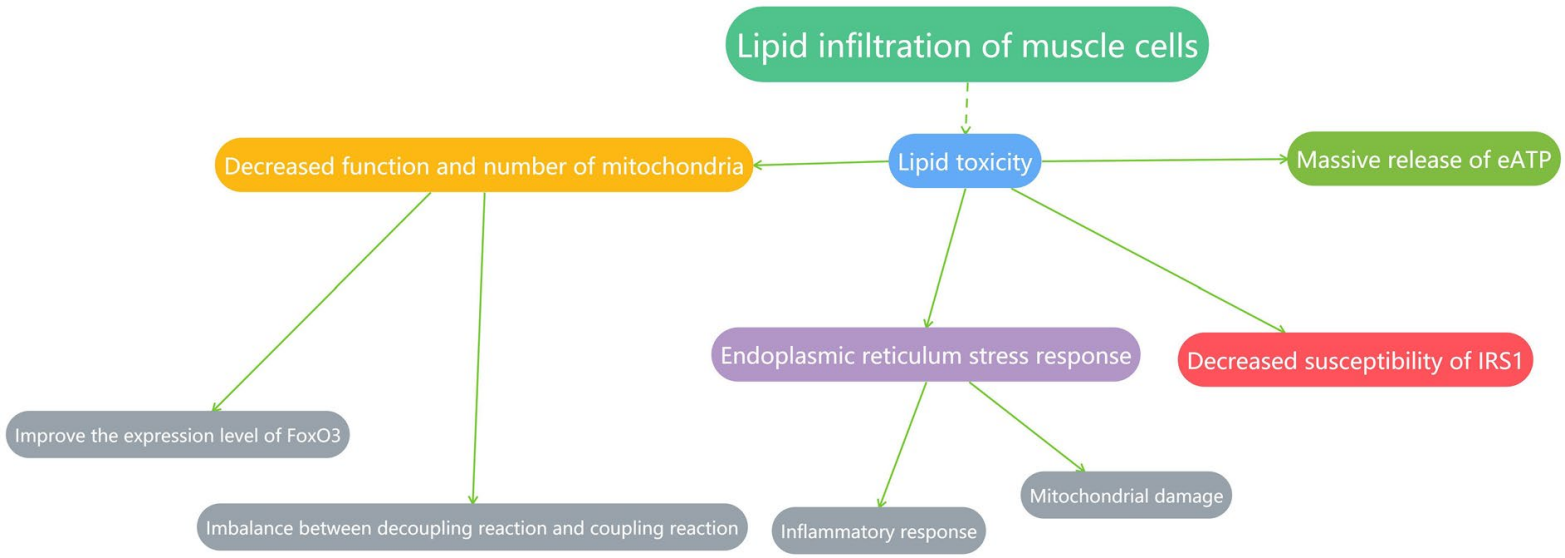
Possible mechanisms by which lipid infiltration of myocytes leads to the development of insulin resistance

The complex relationship between lipid infiltration of myocytes and insulin resistance is a topic of interest, and, in this section, we focus on the effects of lipids on different sites of the insulin action pathway and on organelles and summarize the results of research conducted in recent years (Fig. 3).

### Sarcopenia may lead to increased levels of branched-chain amino acids, causing insulin resistance and ultimately forming a vicious cycle

Increased levels of branched-chain amino acids (BCAA) promote muscle protein synthesis, and evidence suggests that leucine may partially suppress muscle wasting by reducing proteolysis [32]. However, in recent years, increased levels of BCAA and metabolites have been suggested to be associated with the development of insulin resistance.

BCAA are abundant in the muscle, accounting for approximately 30% of muscle protein content. The skeletal muscle is a major site of BCAA catabolism, and branched-chain amino acid transferase 2 (BCAT2) is mainly expressed in the skeletal muscle. Mice with systemic BCAT2 knockout exhibited increased phosphorylation of S6K1 and increased plasma levels of BCAA [93]. Decreased BCAT2 levels in patients with sarcopenia may be one reason for increased levels of BCAA. In addition, the BCAA catabolic pathway may be involved in skeletal muscle protein synthesis and metabolic pathways [68].

In recent years, several studies have focused on the relationship between BCAA and insulin resistance. In this regard, overall, two hypotheses have been proposed: (1) increase in BCAA overactivates MTORC1, leading to hyperactivation of S6K1, which causes dephosphorylation of IRS1 and, consequently, the development of insulin resistance, and (2) accumulation of BCAA leads to mitochondrial dysfunction, which has been elaborated in other reviews [67, 109]. However, the specific relationship between the two and the mechanism of influence are not clear. Different studies have produced different results. The relationship between BCAA and insulin resistance therefore warrants further investigation.

Crossland et al. found that intermediates of BCAA breakdown accumulated significantly after the culture of myotubes in a culture medium with high concentrations of BCAA, and these metabolites inhibited the activity of Akt and ISR1, leading to the development of insulin resistance. BCAA levels were found to decline after promotion of BCAA breakdown with lead however, the levels of BCKA did not change, which may indicate damage to a link in the catabolic pathway of BCAA [23].

Valine is catabolized to produce an intermediate, 3-HIB, a paracrine regulator of transendothelial fatty acid transport. Jang et al. found that endothelial cell trafficking was activated in the presence of 3-HIB, stimulating muscle fatty acid uptake and lipid deposition in the muscle [46]. They tested this conclusion using PGC-1α, a transcriptional coactivator that activates gene expression regulating fatty acid transporters. Inhibition of 3-HIB synthesis in the muscle was found to block the ability of PGC-1α to promote fatty acid uptake in endothelial cells, further demonstrating the role of 3-HIB, a valine catabolic intermediate. 3-HIB can increase the translation level of the fatty acid transporters FATP3 and FATP4, improving fatty acid uptake capacity in endothelial cells and leading to the occurrence of lipid deposition in myocytes. Previous studies have demonstrated that 3-HIB induces an increase in membrane expression of PKC-θ, while decreasing the phosphorylation level of AKT1. Through animal experiments, Giesbertz showed that the serum levels of 3-HIB remained elevated in patients with diabetes in the absence of overexpression of valine catabolic enzymes [41]. Posttranscriptional regulation may lead to abnormal valine breakdown, which ultimately results in the development of insulin resistance through multiple pathways, such as lipotoxicity, lipid infiltration between myocytes, diminished AKT signaling, and decreased insulin signaling receptor sensitivity.

However, BCAA supplementation alone does not induce the development of insulin resistance in regular food-fed rats. However, manifestations of insulin resistance appear in HFD-fed rats. Therefore, controversy exists regarding whether increased BCAA levels can directly lead to the development of insulin resistance or lead to its further development in patients who are already insulin resistant. In a mouse model of insulin resistance induced by fructose feeding, David et al. found that an increase in BCAA levels occurs early, preceding the development of insulin resistance [27]. Other studies have shown that BCAA is unlikely to independently cause the development of insulin resistance [45]. In addition, scientists have observed that increased levels of BCAA in insulin-resistant mice lead to significantly decreased levels of mitochondrial function markers PGC-1α and SIRT1, suggesting that mitochondrial function is compromised and that it may decrease the ability of muscles to catabolize lipids and BCAA. Rivera et al. observed that BCAA reduced mitochondrial metabolic capacity in insulin-sensitive and insulin-resistant cells at supraphysiological concentrations [85].

Asghari et al. followed 1205 Iranian men and women for 2.3 years and showed that dietary intake of high BCAA increased the risk of insulin resistance; however, a limitation of this study was that serum BCAA concentrations were not determined [6].

Hernández-Alvarez et al. showed through clinical studies that T2DM leads to decreased mRNA expression of BCKDHβ and BCAT2 in the skeletal muscle, causing increased levels of BCAA. Defining whether increased levels of BCAA are a secondary consequence of insulin resistance or a predisposing factor is therefore a critical issue. Additionally, future research needs to explore whether primary diseases such as sarcopenia can lead to catabolic disorders of BCAA and, thus, the development of insulin resistance.

### Decreased proportion of type I muscle fibers in the skeletal muscle is an important mechanism inducing insulin resistance

Type I muscle fibers have a higher aerobic metabolic capacity than type II muscle fibers. It has been found that the decrease in the proportion of type I muscle fibers in patients with insulin resistance and diabetes is positively correlated with the severity of insulin resistance, which is an important cause of the development of insulin resistance.

A positive correlation between the concentration of free fatty acids (NEFA) and the degree of insulin resistance has been discussed earlier; it can be shown by objective data from the HOMA homeostasis model, which is positively correlated with results from HOMA-IR and negatively correlated with the proportion of type I muscle fibers. Zhou et al. measured the type of myofibers in HFD-fed mice and found a decreased proportion of type I myofibers, likely due to decreased phosphorylation levels of AMPK because of increased activation of FNIP1 and FLCN. Following the treatment with dihydromyricetin (DHM), TG levels were found to decrease [110] and type II fibers partially shifted to type I fibers as TG levels decreased, further demonstrating that the transition of type I fibers to type II fibers contributes to the development of insulin resistance. Stuart et al. found a significant decrease in the number of type I muscle fibers, more than two-fold increase in the number of type II muscle fibers, and a 60% decrease in whole-body insulin responsiveness, according to the results of lateral thigh muscle biopsies from a large number of patients with insulin resistance,these effects were not proportional to the decrease in the levels of insulin receptor (IRS1) and glucose transporter (GLUT4), indicating that the decrease in the number of type I muscle fibers plays a key role [97].

Type I fibers play an important role in insulin sensitivity because they contain more mitochondria and can undergo more lipid oxidation compared to type II fibers. Another possible mechanism is that the number and density of tiny capillaries surrounding type I fibers is higher than that in type II fibers, possibly because muscle fibers with greater oxidative capacity and selective sensitivity to insulin have a greater number of capillaries, which may alleviate the symptoms of insulin resistance [103]. However, the concentration of insulin receptors (IRS1) is not considerably different between type I and type II muscle fibers. Böhm et al. showed through human experiments that the mitochondria in type II muscle fibers are more vulnerable to changes in the body's metabolic status in patients with obesity. They only found obesity-induced mitochondrial destruction in type II muscle fibers, resulting in impairment of mitochondrial function [15], which may be one reason why an increased proportion of type II muscle fibers leads to insulin resistance [59]. Blackwood et al. proposed a new hypothesis in 2022 that changes in blood glucose stimulate type II skeletal muscle fibers to release an actin that promotes insulin secretion from β-cells, whereas type I muscle fibers do not show this activity; this theory suggests that large areas of type II muscle fibers stimulate excessive insulin release, which is negatively correlated with systemic insulin sensitivity [8, 13, 40]. Experimental evidence is lacking for this hypothesis, and the mechanisms of action underlying myofiber transition is unclear; therefore, this theory can serve as a direction for future research.

## Outlook

The correlation between insulin resistance and sarcopenia has become a hotspot of research. The two can be mutually etiological, resulting in a vicious cycle. The relationship between the two is very complex, involving several molecules and genes. The role of mitochondria in the skeletal muscle in the above two diseases is likely to be an attractive avenue of future research; an increasing number of mechanisms are considered to be related to abnormal mitochondrial function, and good efficacy has been achieved after intervention therapy against mitochondria.

In this paper, we focus on some of the most important mechanisms linking insulin resistance to sarcopenia. In recent years, with the progress of science and technology, experts can perform more in-depth studies to identify pathogenesis mechanisms, such as mutations and deletions at the genetic level, which enrich the connection between the two diseases. However, the impact of a single mechanism is insufficient to explain the pathogenesis of both diseases. Most mechanisms interact with each other and induce further deterioration of the disease. In addition, these two diseases are closely related to various external factors such as age, gender, and amount of exercise, which greatly affect the course of the disease. With the aging of the population, an increasing number of patients will be afflicted with these two diseases; thus, we urgently need to develop practical interventions against the occurrence and progression of insulin resistance and sarcopenia.

## Limitations

The specific mechanisms of insulin resistance and sarcopenia are extensive; thus, the focus of this paper was to elaborate the main mechanisms of their correlation and establish a framework for their connection. In addition, the articles cited herein were sourced from PubMed and may have been incomplete in the elaboration of some mechanisms. Furthermore, the review may have been subjective in the process of literature screening, collation, and analysis.

## Data Availability

Data sharing not applicable to this article as no datasets were generated or analysed during the current study.
